# *Lactobacillus paracasei* HII01 enhances lifespan and promotes neuroprotection in *Caenorhabditis elegans*

**DOI:** 10.1038/s41598-023-43846-9

**Published:** 2023-10-04

**Authors:** Kishoree K. Kumaree, Mani Iyer Prasanth, Bhagavathi Sundaram Sivamaruthi, Periyanaina Kesika, Tewin Tencomnao, Chaiyavat Chaiyasut, Anchalee Prasansuklab

**Affiliations:** 1 Natural Products for Neuroprotection and Anti-Ageing Research Unit, Chulalongkorn University, Bangkok, 10330 Thailand; 2 College of Public Health Sciences, Chulalongkorn University, Bangkok, 10330 Thailand; 3 Department of Clinical Chemistry, Faculty of Allied Health Sciences, Chulalongkorn University, Bangkok, 10330 Thailand; 4 Office of Research Administration, Chiang Mai University, Chiang Mai, 50200 Thailand; 5 Innovation Center for Holistic Health, Nutraceuticals, and Cosmeceuticals, Faculty of Pharmacy, Chiang Mai University, Chiang Mai, 50200 Thailand

**Keywords:** Biochemistry, Preclinical research, Neurodegenerative diseases, Geriatrics

## Abstract

Achieving healthy aging and providing protection from aging-related diseases is a major global concern. Probiotics, are a safer and more natural alternative. Moreover, identifying novel probiotics can help develop a new therapeutic approach and may help in personalized probiotic-formulations for individual's unique gut microbiome. In this study, we evaluated the benefits of our novel probiotic strains in promoting healthy aging and whether they protect against Amyloid β toxicity of Alzheimer's disease. Henceforth, we analyzed the impact of four different probiotics (*Lactobacillus paracasei* HII01, *L. rhamnosus*, *L. reuteri*, *L. salivarius*) on the lifespan extension of *Caenorhabditis elegans* model. Our results determine that *L. paracasei* HII01 provided the most positive effect on longevity and antiaging effects on *C. elegans*. The qPCR data and mutant-based studies indicated that *L. paracasei* HII01-mediated lifespan extension could be modulated by DAF-16 mediated pathway. The probiotic strains also protected the worms from the toxicity induced by β-Amyloid-expressing (Aβ) transgenic *C. elegans* strains, and *L. paracasei* HII01 provided the most significant protection. Overall, identifying novel probiotics is an important area of research that can improve health outcomes. Our study showed that *L. paracasei* HII01 could be considered a dietary supplement for providing healthy aging and preventing aging-related diseases.

## Introduction

The human gut microbiota is a diverse population of bacteria that live in the gastrointestinal system^1^. Alterations in the gut microbiota have been linked to various health issues, including inflammatory bowel disease, obesity, type 2 diabetes, and specific neurological disorders^2–4^. Studies have shown that dietary probiotic supplements are linked to a healthy microbiome and health benefits^5^. Probiotic bacteria are live microorganisms that can promote human health and longevity^6,7^, along with regulating the gut environment and immune regulation^8^. Probiotic strains were reported to produce several health-promoting bioactive components such as enzymes, bacteriocins, short-chain fatty acids, polypeptides, bacteriocin, and neurotransmitters like γ-aminobutyric acid^9–17^.

Moreover, alterations to the eubiotic state of the human gut^18^, which is marked by the presence of diverse microbial populations, undergo potent shifts as the aging process unfolds^19^. Such age-related changes in microbial diversity can be correlated with age-related health issues in the elderly^20,21^. Aging is a consequence of gradual accumulation of detrimental biological factors, resulting in a reduction of structural robustness, and is devised by intricate biological pathways^22^. Aging could also make changes in the gut microbiome, which can have an impact on the gut-brain axis^23^ that could aid in the development of Parkinson's disease (PD), amyotrophic lateral sclerosis (ALS), and Alzheimer's disease (AD)^24^. It has also been shown that altering the dietary pattern to restore the eubiotic state^25^, defined as a balanced and diverse microbial community that supports optimal digestion and immune function, has been associated with improved lifespan among older individuals and a notable reduction in frailty^19,26^. This has given rise to the notion that healthy aging is associated with having healthy microbial diversity^27^.

Several lines of research have indicated a connection between the gut microbiota and central nervous system functioning, known as the gut-brain axis^28^. Alterations in gut microbiota composition have been associated with neuroinflammation, cognitive decline, and the onset of neurodegenerative diseases like AD. Research has demonstrated that individuals who are amyloid-positive or diagnosed with Alzheimer's disease (AD) exhibit distinct alterations in the composition of their gut microbiota (GMB) when compared to those who do not show amyloid positivity or AD^29,30^. Moreover, in vivo*,* studies employing AD-modelled mice have showcased a notable response to various probiotic interventions, demonstrating both direct and indirect impacts on the reduction of amyloid beta (Aβ) levels^31,32^. Although direct conclusive evidence may be lacking between probiotics and neurological health, we intend to contribute to the ongoing exploration of the potential therapeutic applications of probiotics, including Lactobacillus strains, in mitigating neurodegenerative diseases like AD.

*Caenorhabditis elegans* is an excellent experimental model for studying several biological processes like obesity^33^, toxicology^34^, immune response^35^, host–pathogen interactions^36^, aging^37^ and neurodegenerative diseases^38^. The microbiome of *C. elegans* has crucial implications for understanding the interaction between hosts and their microbial ecosystems. Previous reports suggest that *C. elegans* can be used as an ideal model to study the benefits of probiotic supplementation^39,40^. Using *C. elegans* as an experimental model, different probiotic strains were reported to have beneficial effects, including healthy aging, and aging-related factors like leaky gut, inflammation, oxidative stress, and irritable bowel syndrome (IBM) have been reported earlier^41–43^. It was reported that supplementing the food of *C. elegans* with *L. plantarum*-JBC5 led to an increase in gut integrity by activating intestinal tight-junction-protein ZOO-1 and improved cognitive response by activating the gene responsible for serotonin signaling^44^. However, the underlying processes still need to be fully understood. Hence, before extrapolating to humans, the importance of gut colonization of a probiotic strain to have its best effects on worms must be further studied.

Herein we explored the dynamics of gut microbiome-host interaction and its relevance to aging. This comparative study involved four lactic-acid bacteria (LAB) strains, earlier isolated and reported to have probiotic properties and exhibited beneficial health benefits^45,46^. Henceforth, the current study investigates whether probiotics could extend the lifespan of *C. elegans* and delay aging-related physiological parameters. Furthermore, we also aimed to analyze the underlying molecular mechanism elucidated by these probiotics in the lifespan extension of *C. elegans*.

## Results

### Lactobacilli feeding extends the lifespan of *C. elegans*

**Figure 1:**
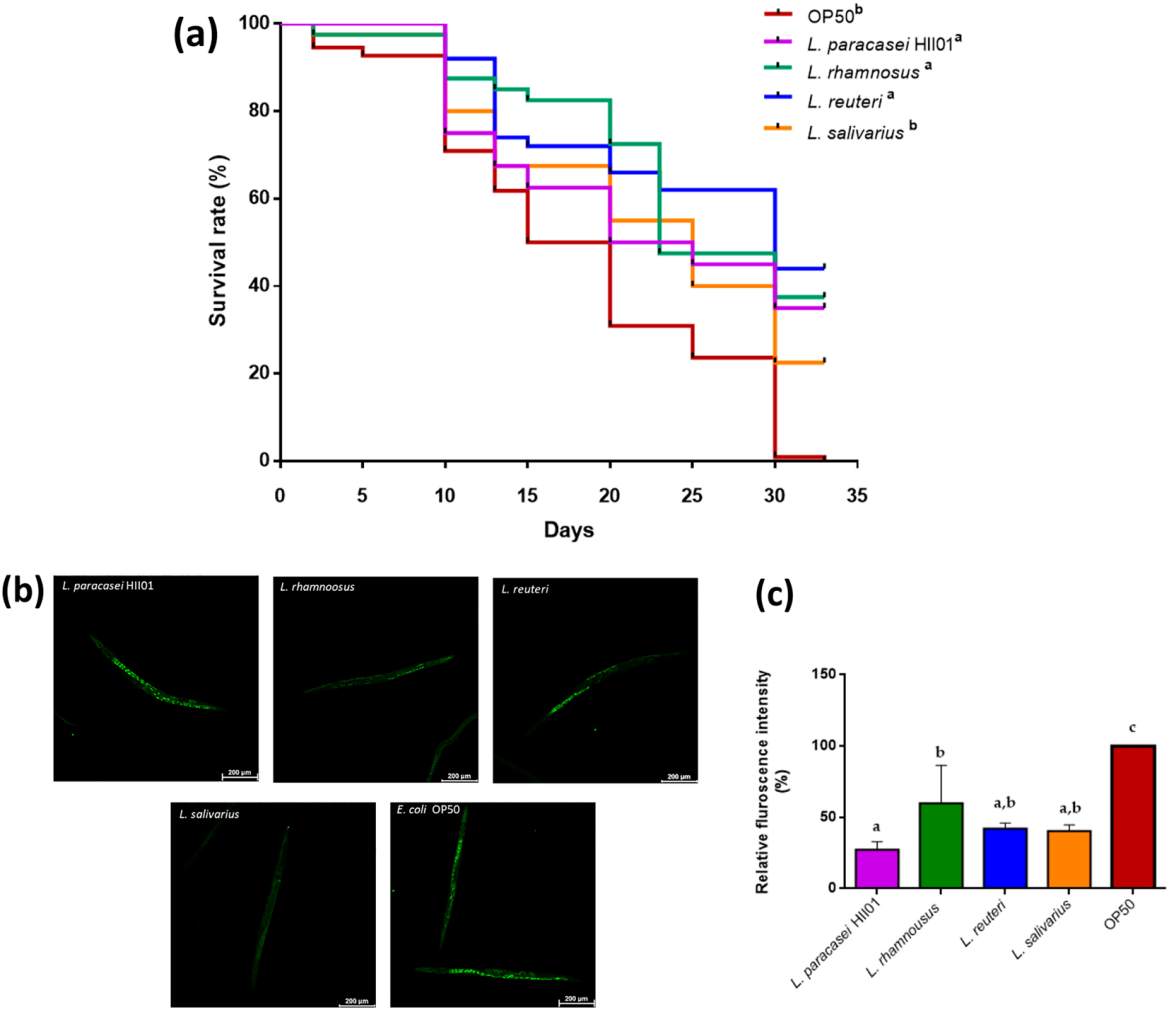
Survival analysis and lipofuscin accumulation of the worms fed with Lactobacillus probiotics or *E. coli* OP50. (**a**) The survival curve of the worms fed with OD_600_ 0.5 of *L. paracasei* HII01, *L. rhamnosus*, *L. reuteri*, *L. salivarius,* and control worms fed with *E. coli* OP50. Significance refers to four LAB strains' effect on worms fed with control OP50. Data were obtained through three independent experiments. Kaplan–Meier survivorship curves over time (days) for *C. elegans* were plotted with the log-rank (Mantel-Cox) statistical test to compare the experimental groups. Different colored lines represent the lifespan curve of worms fed with individual Lactobacillus probiotics. (**b**) Lipofuscin accumulation in wild-type *C. elegans* (N2) fed with *E. coli* OP50 or LAB strain (OD_600_ 0.5). After day 5, lipofuscin was measured by assessing autofluorescence using the confocal microscope. Representative lipofuscin fluorescence images in worms fed with LAB strains; *L. paracasei* HII01, *L. rhamnosus*, *L. reuteri,* *L. salivarius* and *E. coli* OP50 on day 5. Images were captured at 10X magnification through confocal microscopy. (**c**) Bar graph showing one-way ANOVA for the florescence quantified using ImageJ software (mean ± SD). Different superscript letters indicate statistically significant differences among the groups (*p* < 0.05).

Feeding different strains of *Lactobacillus* at OD_600_ 0.5 increased the overall survival of *C. elegans* (N2) (Fig. 1a) compared to *E. coli* OP50*.* Different doses of each *Lactobacillus* were used to determine the optimum dose, and the results showed that the studied strains could increase the lifespan dose-dependently (Supplementary Fig. S1). The lifespan was significantly extended in worms fed with *L. paracasei* HII01*, L. rhamnosus,* and *L. reuteri* when given the higher dilution of the bacterial culture (OD_600_ 0.5) compared to the control worms fed with *E. coli* OP50. However, worms given OD_600_ 0.3 and 0.1 of *L. paracasei* HII01 had no significant difference compared to the control group (Supplementary Fig. S1). For *L. rhamnosus,* all three doses significantly improved compared to the control group. However, for *L. salivarius* all three doses chosen did not show any significant changes from the control group *C. elegans* (N2) (Supplementary Fig. S1). Figure 1a summarizes the dose of OD_600_ 0.5 for all the LAB strains, compared to *E. coli* OP50, *L. paracasei* HII01, *L. rhamnosus,* and *L. reuteri* had a significant (*p* < 0.05) effect on the lifespan of *C. elegans* (N2). Therefore, the OD_600_ 0.5 dose was chosen for further experiments based on the lifespan curves.

Among various biomarkers, lipofuscin accumulation stands as a notable indicator within the spectrum of aging processes. The worms fed with *L. paracasei* HII01 had the lowest detection of lipofuscin compared to other strains and *E. coli* OP50 (Fig. 1b,c). In contrast, worms fed with *L. rhamnosus, L. reuteri,* and *L. salivarius* had no significant difference within the group but were significantly different from *E. coli* OP50. Interestingly, *L. salivarius* reduced lipofuscin accumulation in worms while exhibiting minimal impact on their longevity. This phenomenon indicated that *L. paracasei* HII01 consumption could reduce the accumulation of aging pigment the most compared to all the treatments. The colony forming unit (CFU) of each *Lactobacillus* strain was detected in the intestine of the worms until day three. However, no significant difference was found among the *Lactobacillus* strains (Supplementary Fig. S2). A similar trend was seen for the worms' probiotic preferences during the chemotaxis assay. As mentioned in Table 1, all the probiotic strains were equally preferred by the worms and showed no significant difference in their chemotaxis indices.

**Table 1:** Chemotaxis index (CI) between four LAB strains at 3 and 6 h (OD_600_ 0.5).

Combination of LAB strains comparison		Index hour 3^a^	Index hour 6^a^
1	*L. paracasei* HII01 *L. rhamnosus*	−0.086 ± 0.173	−0.226 ± 0.172
2	*L. paracasei* HII01 *L. reuteri*	0.000 ± 0.009	0.094 ± 0.092
3	*L. paracasei* HII01 *L. salivarius*	−0.201 ± 0.187	−0.289 ± 0.201
4	*L. rhamnosus* *L. reuteri*	−0.125 ± 0.218	0.025 ± 0.103
5	*L. rhamnosus* *L. salivarius*	0.155 ± 0.393	0.089 ± 0.165
6	*L. reuteri* *L. salivarius*	−0.034 ± 0.168	−0.122 ± 0.109

^a^The values are mean ± SD. The positive number of the CI signifies the preference of worms for the strain at the numerator and vice versa for the negative index number.

### Lactobacillus probiotics enhanced mitochondrial function.

**Figure 2:**
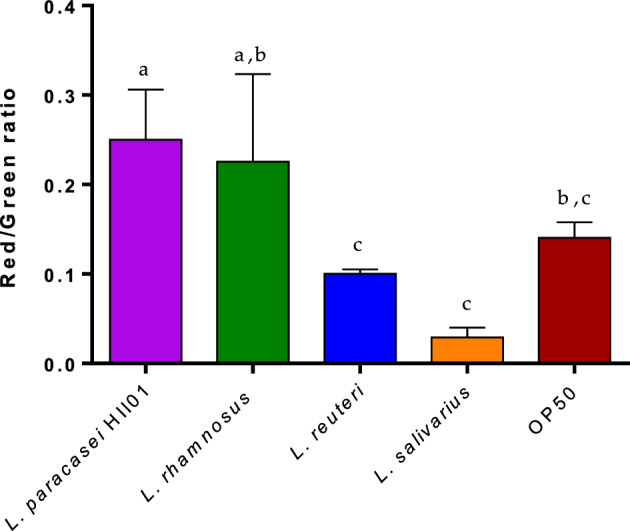
Investigation of red or green fluorescence intensity by JC-1 staining. After 5 days red/ green fluorescence was examined using JC-1 dye staining for worms treated with individual LAB strains (OD_600_ 0.5) or *E. coli* OP50, bar graph showing one-way ANOVA for the red/green fluorescence (mean ± SD). Different superscript letters indicate statistically significant differences among the groups (*p* < 0.05).

The above experiment showed that changing the diet to our LAB strains benefits the worms' aging. Mitochondrial oxidative stress due to defective mitochondria adversely affects overall physiology, contributing to aging and age-related diseases; hence, approaches to alleviate this stress hold promise as a therapeutic strategy. So, to gain further insights into mitochondrial ROS production, the assessment of mitochondrial membrane potential was performed utilizing the cyanine dye JC-1. JC-1, a green-colored dye, accumulates within mitochondria leading to the formation of JC-1 aggregates (red), indicating healthy mitochondrial membrane potential. The alteration in the ratio of red/green fluorescence serves as an indicator of mitochondrial health, reflecting both mitochondrial membrane (ΔΨm) and ROS status^47^. In our study, we observed that wild-type worms fed with *L. paracasei* HII01 displayed significantly higher (*p* < 0.01) red fluorescence levels in comparison to those fed with *E. coli* OP50 (Fig. 2). In contrast, other treatments did not improve compared to the control group.

### Lactobacillus paracasei HII01 regulated insulin/IGF-1 signaling (IIS) pathway and SKN-1

**Figure 3:**
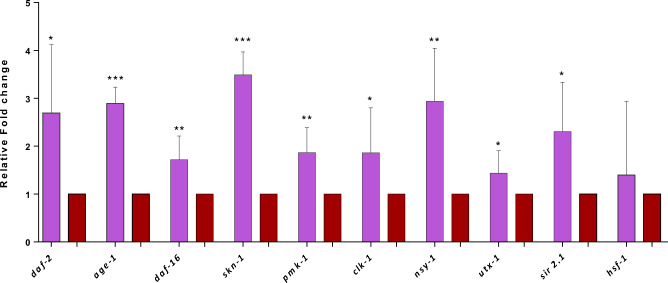
The expression of lifespan extension-related gene changes in the wild-type *C. elegans,* in response to feeding *L. paracasei* HII01 only (OD_600_ 0.5). Significant changes are relative to *E. coli* OP50. The expression level of each gene was normalized to that of *act-2*. The error bars indicate the standard deviation (SD). Each experiment was performed with three replicates, and the sample comprised of 1000 ng of cDNA converted from total RNA for each treatment group. *p < 0.5, ** *p* < 0.01, and ***p < 0.001 (Independent samples t*-*test).

**Figure 4:**
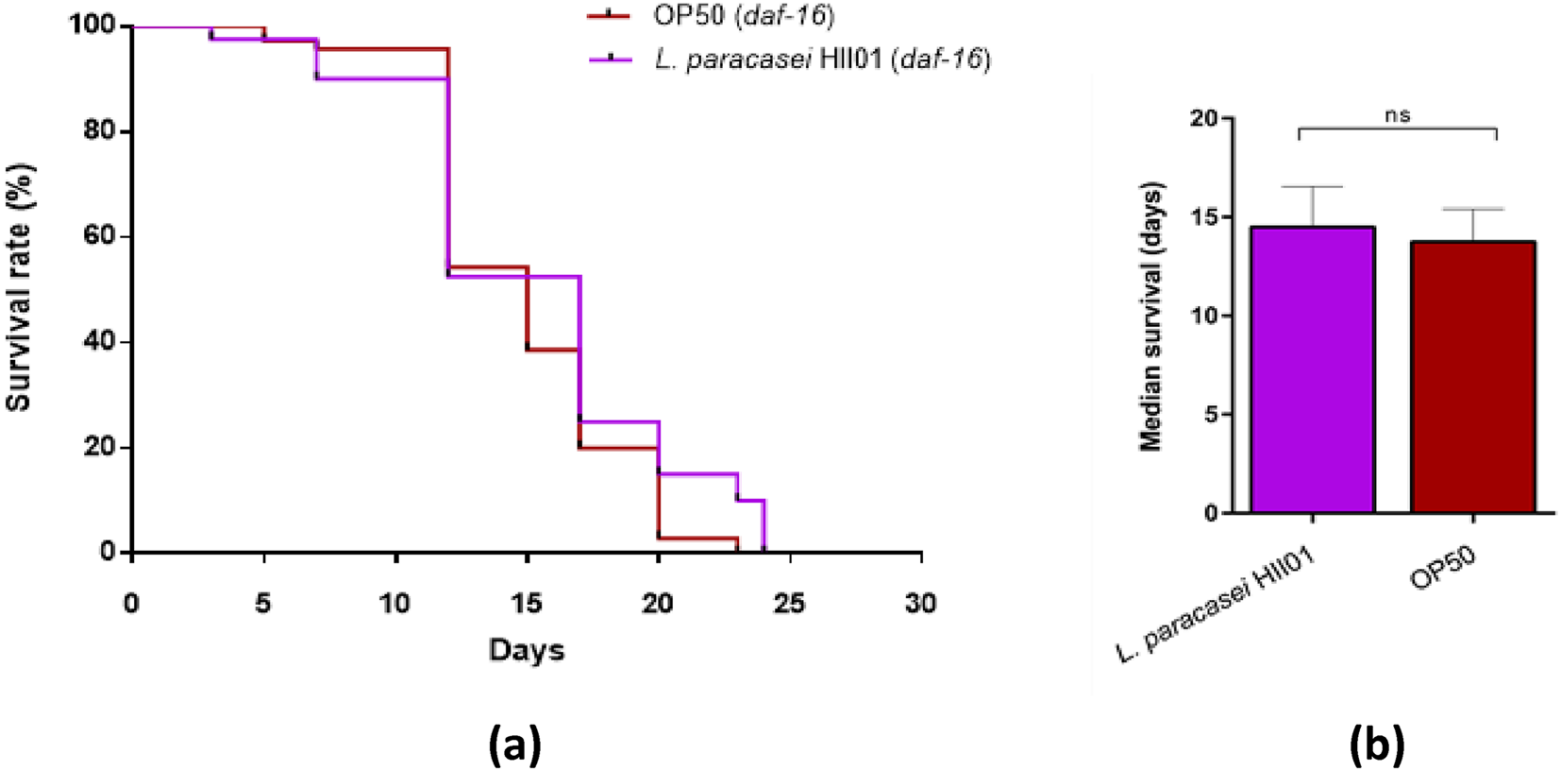
The effect of *L. paracasei* HII01 (OD_600_ 0.5) feeding on the overall lifespan of *C. elegans* mutant *daf-16* (CF1038). (**a**) Kaplan–Meier survivorship curves over time (days) for *C. elegans* (*daf-16* mutant) were plotted with the log-rank (Mantel-Cox) statistical test to compare the worms fed *E. coli* OP50 or *L. paracasei* HII01. The survival rate was recorded in regular intervals. (**b**) Median survival (in days) of control worms fed on *E. coli* OP50 and *L. paracasei* HII01-fed mutant worms. The error bar represents SD. All experiments were carried out in duplicates and repeated two times. NS meant that the difference was statistically not significant.

We selected *L. paracasei* HII01, based on the previous results, to evaluate the underlying pathways associated with *Lactobacillus-*mediated longevity. The qPCR analysis was conducted using genes associated with the longevity and stress response (mentioned in Supplementary Tables S1 and S2) to investigate the expression of those genes in *C. elegans* when fed with *E. coli* OP50 and *L. paracasei* HII01 of OD_600_ 0.5. The relative fold changes of *daf-2, age-1, and daf-16* were upregulated significantly *(p* < 0.05) compared to the *E. coli* OP50 (Fig. 3). The expression levels of *pmk-1 and nsy-1* were significantly (*p* < 0.05) upregulated in worms fed with *L. paracasei* HII01 compared to *E. coli* OP50. In addition, there was a significant increase in the gene expression of *skn-1*, *clk-1* (mitochondrial polypeptide), and *sir-2.1* (sirtuin deacetylase). The survival rate of the mutant worms *daf-16* (CF1038) was monitored to elucidate the involvement of IR and FOXO (forkhead box-O transcription factors) in the longevity of worms fed with *L. paracasei* HII01. *C. elegans* mutants in *daf-16* fed with *L. paracasei* HII01 had no significant changes in their lifespan compared to the control worms (Fig. 4).

### Effect of Lactobacilli on the transgenic strains of C. elegans expressing Aβ peptide

**Figure 5:**
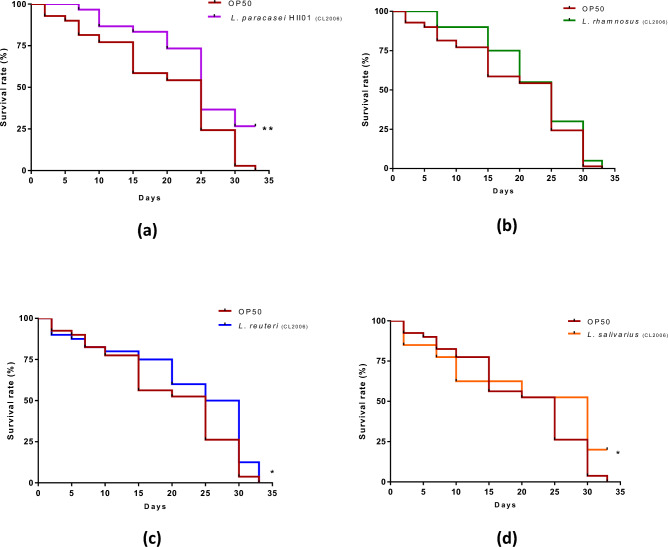
Kaplan–Meier survivorship curves over time (days) for *C. elegans* (CL2006) were plotted with the log-rank (Mantel-Cox) statistical test to compare the worms fed with four different LAB strains (OD_600_ 0.5) or *E. coli* OP50. Age-synchronized worms were fed with *E. coli* OP50 or individual LAB strains. (**a**) *L. paracasei* HII01, (**b**) *L. rhamnosus*, (**c**) *L. reuteri* and (**d**) *L. salivarius*. *p < 0.05 and **p < 0.01.

It was hypothesized that there could be possible protection against neurodegenerative diseases (particularly AD) due to the implementation of our probiotics. Therefore, four of our probiotics were provided to the transgenic *C. elegans* strain (CL2006), which expressed human amyloid beta (Aβ) peptide, a marker protein for AD pathology. Figure 5 shows the comparative analysis of the survival curve of all four lactobacilli. It clearly showed that *L. paracasei* HII01 provided the maximum protection to the transgenic worms and could significantly improve the overall lifespan, followed by *L. reuteri* and *L. salivarius*, whereas *L. rhamnosus* had no significant protection against Aβ toxicity in the worms compared to the worms fed with *E. coli* OP50.

## Discussion

As we age, our microbiome also undergoes transition; such changes in the microbiota are mostly correlated with health-related outcomes in the elderly^19,48^. The gut harbors trillions of diversified bacterial cells to produce an effective micro-environment^49^, and it behaves like a second brain, significantly contributing to various physiological conditions of human health. Therefore, alteration in probiotic intake can eventually change intestinal microbiota, impacting the overall aging process in adults^50^. Ikeda et al. reported various probiotic (*Bifidobacterium, Lactobacilli*) applications in *C. elegans* involving anti-aging effects^51^. To test this hypothesis that the consumption of probiotics can improve the aging process, we determined the impact of the consumption of four different lactobacilli on the aging process of *C. elegans.* Moreover, the probiotic bacteria' mechanism in prolonging the lifespan of nematode still needs to be completely understood. So, in the present study, our findings suggest that *C. elegans* is a useful in vivo model to evaluate the potential LAB as enhancers in several physiological activities and improve the overall aging process.

In this study, we found that feeding four different *Lactobacilli* can improve the lifespan of *C. elegans* in a dose-dependent manner and other parameters related to healthy aging. Based on our initial comparative analysis of all the probiotics, it was found that there was no significant difference amongst the groups of *C. elegans* fed with *L. paracasei* HII01, *L. reuteri* and *L. rhamnosus,* however *L. salivarius* had no significant changes in lifespan contribution compared to the control worms fed with *E. coli* OP50. Building on these findings, subsequent assays were undertaken in an effort to identify a better probiotic candidate that could provide enhanced efficacy across multiple assessments aimed for contributing to healthy aging. Feeding worms with *L. paracasei* HII01 showed the most reduction in lipofuscin accumulation compared to all the probiotics (Fig. 1B). Our findings imply that different species of *Lactobacilli* have varying biological impacts, which has led to the different patterns of aging in *C. elegans*. The present result agreed with studies showing that a probiotic *Propionibacterium freudenreichii* fed to *C. elegans* showed reduced lipofuscin accumulation^44,52,53^.

Having a healthy gut is one of the major contributions to healthy aging^54^, contributing to resistance to other pathogenic bacterial proliferation. For such conditioning, it was essential to know if the probiotics in this study could help colonize the intestine of the nematodes. From the bacterial burden assay (Fig. S2), it was evident that probiotics could be successfully found in the worms for up to the next three days, even though worms were deprived of any feed source. Importantly, all four LAB strains had a successful proliferation. However, there was no significant difference detected amongst strains. Findings in this study were per the study conducted by Kim et al*.*, who demonstrated that *C. elegans,* when conditioned with *L. acidophilus*, failed to colonize the intestine of the nematodes; however, it provided a significant reduction in the infection of *Enterococcus faecalis*^55^. Hence, it would be essential to know the protective effect of our four LAB strains against pathogenic bacterial proliferation, and further experiments can be suggested to validate further the beneficial impacts of our probiotic isolates.

DAF-2 acts as an antagonist to DAF-16 translocation in the DAF-2/DAF-16 pathway, where the gene expression of *daf-16* is reduced hence shortening the lifespan of the worms. DAF-16 regulates the genes involved in adult longevity, stress resistance, and antimicrobial response^56^. In our study, the gene expression at mRNA levels shows the upregulation of *daf-2* and *daf-16*; moreover, *L. paracasei* HII01 failed to prolong the lifespan of *daf-16* mutants (Fig. 4). This suggests the involvement of DAF-16 in the lifespan extension of the worms. Oh et al. reported that the c-Jun N terminal kinase (JNK) family, a subgroup of the MAPK signaling pathway, is a positive regulator of the activity of DAF-16^57^*.* It could be possible to state that *L. paracasei* HII01 enhanced the host lifespan by activating DAF-16 through the JNK pathway. Further experiments are needed to validate the involvement of the JNK pathway and its interconnection with other pathways in the lifespan extension of *C. elegans* due to probiotic feeding. Interestingly, a recent study reported the activation of DAF-16, which aided in the antioxidant mechanism and is independent of the DAF-2 or JNK pathway^58^.

Mitochondrial function degrades with aging, and MMP becomes more depolarized. This depolarization is expected to increase reactive oxygen species (ROS) formation and decreased cell resistance to stress^59^. This study shows that there could be an improvement in MMP in *L. paracasei* HII01-fed worms compared to control worms and other probiotic-fed worms (Fig. 2), which is also supported by our qPCR analysis. However*, L. salivarius* and *L. reuteri*, did not show any significant changes to the mitochondrial membrane potential, compared to OP50. The inconsistency between the two assays highlights the complex nature of microbial interactions and aging-related outcomes. It's important to consider that different probiotic strains may exert varying effects on aging pathways, leading to complex outcomes in different assays^60^. In light of these findings, further investigation is warranted to explore molecular interactions between probiotics and their host, in understanding their potential in influencing healthy aging. Additionally, current days research mainly focuses on using drugs that could target mitochondrial activity as a possible therapy for age-related disorders. In contrast, our study proves the presence of a safer and natural intervention that could potentially enhance mitochondrial function and may have the potential to improve the aging process. Considering the complex interactions among various lactobacilli strains and their combined effects, investigating the potential synergistic effects of a combined lactobacilli strain cocktail could provide valuable insights, the extensive nature of such an experiment warrants future exploration to uncover possible interactions among the diverse strains employed in this study.

In our qPCR analysis, genes that play pivotal roles across diverse molecular pathways intricately linked to aging and longevity were included. These genes were carefully chosen based on their established significance in processes such as insulin/IGF-1 signaling (*age-1*, *daf-2*, *daf-16*), transcription factors (*hsf-1 and skn-1*), oxidative stress response, and mitochondrial function (*clk-1, sir-2.1, utx-1*), additionally, the exploration was extended to genes associated with stress signaling or p-38/MAPK pathway (*pmk-1, nsy-1*)^61–68^. The p38/MAPK pathway has a significant role in inflammation and immune modulation, a hallmark of aging^69^. Representative genes belonging to this pathway that were targeted in this study (*pmk-1*, *nsy-1, skn-1*)^52^ were found to have significantly upregulated (Fig. 3), indicating that *L. paracasei* HII01-fed worms have an enhanced p38/MAPK signaling pathway. Simultaneously, we also observed that *L. paracasei* HII01 feeding to the worms could induce the transcription of an antioxidant gene related to the SKN-1 activation like the expression of *clk-1* was significantly upregulated, whereas *hsf-1* (heat shock protein) was non-upregulated considerably. CLK-1 has a direct correlation with mitochondrial function; it was reported earlier that the overexpression of the *clk-1* gene was associated with enhanced mitochondrial activity^70^.

A significant consequence of aging is an increased risk of developing neurodegenerative diseases such as Alzheimer's, Parkinson's, and Huntington's disease. Alzheimer's disease is characterized by abnormal accumulation of Aβ in plaques. Supposedly, averting the deposition of Aβ oligomers and reducing oxidative stress could reduce the onset of AD. Transgenic *C. elegans* expressing human Aβ-(CL2006) is a valuable model for understanding and screening in vivo drugs for AD treatment. In the present study, CL2006 worms were fed on four of our LAB strains and found the highest lifespan extension in the transgenic worms when fed on *L. paracasei* HII01 compared to the control worms fed with *E. coli* OP50. The other two strains, *L. reuteri* and *L. salivarius,* significantly changed lifespan enhancement. It is interesting to know that the same genera of lactic acid bacteria have a diversified contribution to the lifespan of worms. The lifespan extension of CL2006 was earlier reported by Du et al*.* and linked to worms' decreased ROS content or increased antioxidant content due to Zijuan Pu'er tea water extract treatment^71^; this could explain the varying protective effects of diverse LAB strains. Conducting qPCR analysis on Aβ deposition in Aβ-mutants (CL2006) could provide knowledge on the underlying mechanism of whether bacterial colonization has indeed influenced Aβ levels, providing a more comprehensive understanding of the role of LAB strains in mitigating age-related neurodegenerative processes. Even though, not addressed in our current study, the investigation of Aβ peptide regulation in response to lactobacilli colonization holds promise for future research.

**Figure 6:**
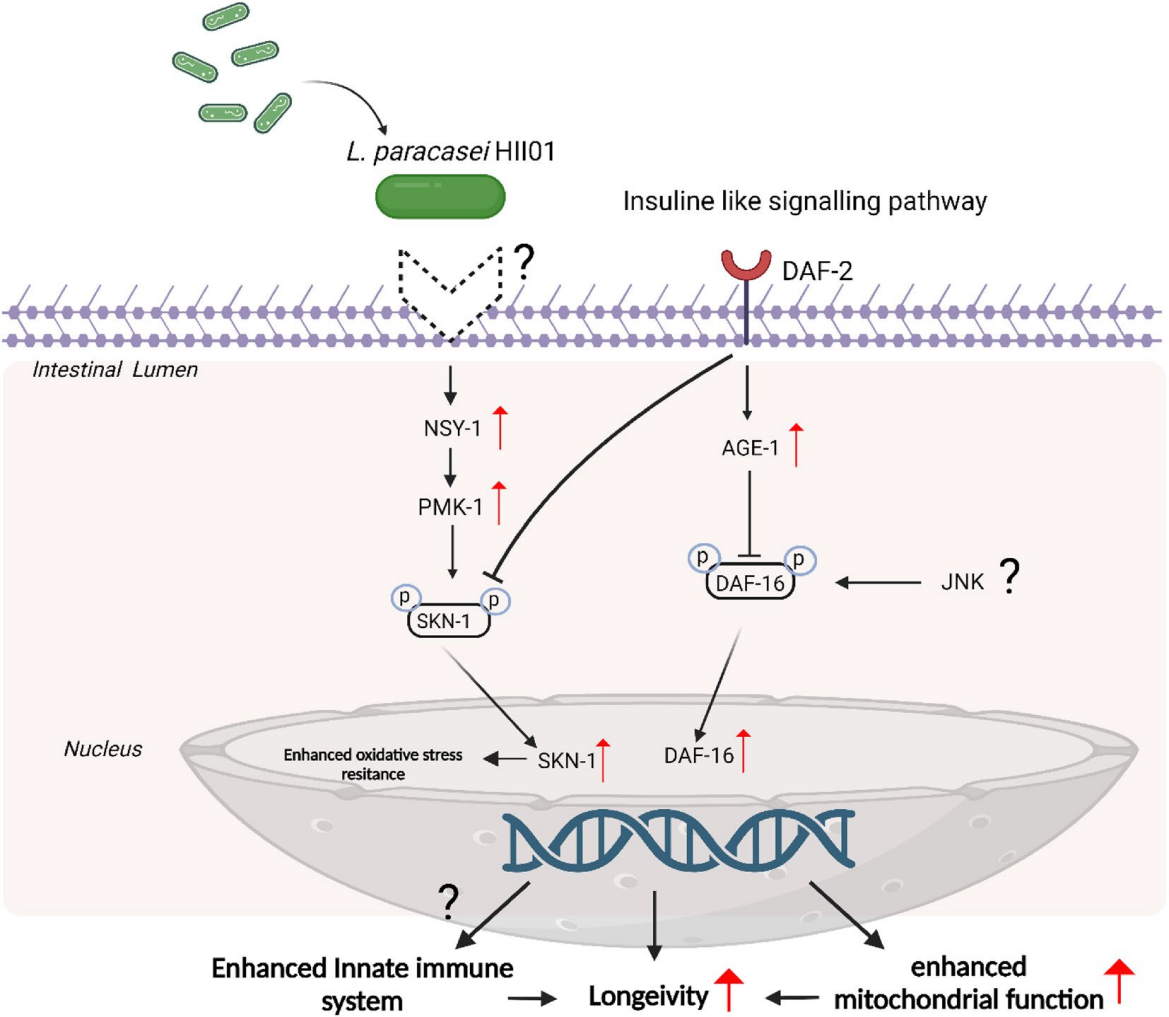
Schematic representation of the mechanism by which *L. paracasei* HII01 affects longevity in *C. elegans*. *L. paracasei* HII01 increased longevity by activating the DAF-16 signaling pathway and its target genes which eventually induced antioxidant molecules, and preventing the reduction of mitochondrial functions such as increasing membrane potential. Overall, *L. paracasei* HII01 increased longevity and enhanced mitochondrial function in *C. elegans.*

In conclusion, this study shows that all our LAB strains have a healthy contribution. Amongst them, *L. paracasei* HII01 was the most effective in providing longevity and improving other aging-related parameters in *C. elegans* (Fig. 6). The findings suggest that *L. paracasei* HII01 could extend the lifespan of *C. elegans*, probably through DAF-16 mediated pathway. *L. paracasei* HII01 significantly protected the worms expressing Aβ, a marker protein for AD. This study also opens opportunities for future investigation on how probiotics could reduce the manifestation of neurodegenerative diseases. Aging is a complex phenomenon driven by diverse factors, and it would not be wrong to state that the proper type of diet could positively reshape human health.

## Materials and methods

### *Caenorhabditis elegans* strains and their maintenance

The different *C. elegans,* such as the wild-type N2 (Bristol), daf-16 mutant CF1038, and Aβ transgenic strain CL2006, were used and procured from *Caenorhabditis* Genetics Center, University of Minnesota. Worms were synchronized by extracting eggs in sodium hypochlorite-sodium hydroxide solution and transferring them onto nematode growth medium (NGM) plates seeded with *E. coli* OP50 and grown at respective temperatures to conduct further experiments per standard procedure^72^.

### Probiotics and their culture preparation

*Lactobacillus* strains were kindly given for this study by Dr. Chaiyavat Chaiyasut (Chiang Mai University, Thailand), namely *Lactobacillus paracasei* HII01 (*L. paracasei* HII01), *Lactobacillus rhamnosus* (*L. rhamnosus*), *Lactobacillus reuteri* (*L. reuteri*), and *Lactobacillus salivarius* (*L. salivarius*). The strains were selected based on their human origin and other probiotic attributes (resistance to acidic environment, non-pathogenic nature, mucus adherence property, and antimicrobial peptide production). First, strains were inoculated into 1% De Man, Rogosa, and Sharpe (MRS) broth (HiMedia, India) and incubated for 18 h at 37 °C. Then, the probiotic cells were harvested by centrifugation at a speed of 5000 × g for 10 min at 4 °C, followed by washing twice with 0.9% normal saline solution. Finally, the cells were resuspended in a similar buffer volume. The harvested cells were diluted into three different OD_600_ (0.5, 0.3, and 0.1), and three of these doses were used for the preliminary screening and defining the optimum working dose for each probiotic.

### Lifespan assay

*Caenorhabditis elegans* lifespan assay was performed using the method described previously with slight modifications^73^. The lifespan assay was determined to evaluate whether the candidate probiotics impact *C. elegans*. Lifespan measurements were performed in 24 welled plates in liquid media. Age-synchronized worms (wild-type or mutants) were cultured till they reached the young adult stage, ten worms were transferred to each well of a 24-well plate containing individual LAB culture (OD_600_ 0.5, OD_600_ 0.3, and OD_600_ 0.1 of each probiotic) or *E. coli* OP50, along with 5-Fluoro-2’-deoxyuridine (FUDR), to prevent progeny production, in every well. Worm survival was recorded at regular intervals, and worms that did not react to the mechanical stimulation were considered dead. At least three independent replications were performed for each lifespan assay. Worm survival was analyzed by the Kaplan–Meier method using GraphPad Prism 8.0, and the log-rank test (Mantel-Cox) was used to detect a significant difference between experimental treatments.

### Chemotaxis assay

A chemotaxis assay investigated the preference for LAB strains among the worms. The assay was done as previously mentioned but with slight modifications^74^. Briefly, 20 µl of each overnight culture LAB strain was seeded at an equal distance from the center of a 5 cm Petri dish, followed by releasing approximately 50–60 worms at the center of the petri dish. A two-quadrant system was followed, where the worms' movement was monitored equidistant from the test subjects. The number of worms was checked after two h and 6 h of incubation at 20 °C. The assay was performed in triplicates. For every assay, a chemotaxis index (CI) was calculated using the following formula:

$$\mathrm{Chemotaxis\, index }(\mathrm{CI}) = \frac{(No.\, of\, worms\, at\, strain\, A -No.\, of\, worms\, at\, strain\, B)}{Total\, number\, of\, worms\, (Initial)}$$

The positive ( +) CI indicates the affinity of worms for LAB strain A was higher than strain B, whereas the negative (-) CI index suggests the attraction of worms more towards strain B.

### Measuring the bacterial burden in the *C. elegans.* intestinal tract

The number of probiotic colonies in the worm intestines was determined according to the modified method^55^. The wild-type worms (L4 stage) were collected and washed (M9 buffer) before being transferred to a medium containing individual LAB cells and incubated for 24 h at 20 °C. After the incubation, worms were washed and transferred into new wells (100 worms) with M9 medium only. Additionally, worms were deprived of an additional food source until the end of the experiment. For determining the bacterial burden, 20 worms were collected on days one, two, and three, washed multiple times, transferred 100 µL of buffer (a sterile tube), and disrupted mechanically using a micropestle. The supernatant of the homogenized solution was further diluted and plated onto MRS agar plates and incubated at 37 °C overnight.

### Fluorescence imaging of age pigment (Lipofuscin)

The lipofuscin accumulation in wild-type N2 worms was determined at day five after feeding with OP50 or LAB strains. Approximately 200 worms of the L4 stage were treated with individual probiotics (OD_600_ 0.5) and OP50 separately in 6 well plates. Worms were removed from the liquid media, washed with M9 buffer, and transferred to a glass slide containing sodium azide. Fluorescence was captured using a Fluoview FV10i (OLYMPUS, Japan) confocal microscope under 10X magnification. Lipofuscin levels were measured using ImageJ software (NIH Image) and determining the average pixel intensity for each worm.

### JC-1 staining

Mitochondrial membrane potential was determined using JC-1 dye (Thermo Fisher Scientific), with slight modifications from the previously mentioned protocol^75^. Approximately 100 worms were given the treatment of individual probiotics and OP50 separately in 24 well plates. On day five, worms from different treatments were collected and washed three times with phosphate-buffered saline (PBS) buffer. Worms were resuspended in 100 µL of PBS, and JC-1 dye (10 µM) was added to each tube and incubated at 37 °C for one hour. Samples were washed three times and resuspended into 96-well black plates (ten worms per well). Measurement of red fluorescence (ex 550 nm/em 600 nm) and green fluorescence (ex 485 nm/em 535 nm) was done using a fluorescence plate reader (Enspire® Multimode Plate Reader; Perkin-Elmer).

### RNA extraction and qPCR analysis

After five days of treatment, total RNA from wild-type worms was extracted using the Trizol method (Invitrogen, Carlsbad, CA, USA). 1000 ng of total RNA was taken for reverse transcription using Accupower RT Premix (Bioneer, Korea) for first-strand cDNA synthesis, following the manufacturer's protocol. The quantitative real-time (qPCR) analysis was carried out on an Exicycler Real-Time Quantitative Thermal Block (Bioneer) with SYBR green and Green Star PCR Master Mix (Bioneer). The relative gene expression level was calculated using the 2^-ΔΔCt^ method^76^, keeping *act-2* as a housekeeping gene to normalize the gene's expression data. Primers used in this assay are mentioned in Supplementary Table S1 and the gene descriptions are mentioned in Supplementary Table S2.

### Statistical analysis

SPSS statistical software for Windows (version 22.0, Armonk, NY; IBM Corp.) was used to analyze the data. Worm survival was analyzed by the Kaplan–Meier method, and the log-rank test was used to detect a significant difference between experimental treatments. An ANOVA was carried out, and Tukey's multiple comparisons were employed to test for significant differences between the means of different doses of LABs. Furthermore, the student's t-test was used for the significance comparison between *E. coli* OP50 and LABs strains. The *p*-value < 0.05 was considered to differ significantly.

### Supplementary Information

Supplementary Information.

## Data Availability

The data generated or analyzed during the current study that are relevant to the results presented here have been included in this article and its supplementary information file.

## References

[1] Clemente JC, Ursell LK, Parfrey LW, Knight R (2012). The impact of the gut microbiota on human health: an integrative view. Cell.

[2] Dinan TG, Cryan JF (2017). The microbiome-gut-brain axis in health and disease. Gastroenterol. Clin..

[3] Zhang X (2013). Human gut microbiota changes reveal the progression of glucose intolerance. PloS one.

[4] Pascal V (2017). A microbial signature for Crohn's disease. Gut.

[5] Zmora N, Suez J, Elinav E (2019). You are what you eat: diet, health and the gut microbiota. Nat. Rev. Gastroenterol. Hepatol..

[6] Metchnikoff, I. I. *The prolongation of life: optimistic studies*. (Springer Publishing Company, 2004).

[7] Pan M, Kumaree KK, Shah NP (2017). Physiological changes of surface membrane in Lactobacillus with prebiotics. J. Food Sci..

[8] Zheng D, Liwinski T, Elinav E (2020). Interaction between microbiota and immunity in health and disease. Cell Res..

[9] Tang C, Lu Z (2019). Health promoting activities of probiotics. J. Food Biochemi..

[10] Hegarty, J. W., Guinane, C. M., Ross, R. P., Hill, C. & Cotter, P. D. Bacteriocin production: a relatively unharnessed probiotic trait? *F1000Research* **5** (2016). 10.12688/f1000research.9615.1 PMC5089130 27853525

[11] Bodera P, Chcialowski A (2009). Immunomodulatory effect of probiotic bacteria. Recent Patents Inflam. Allergy Drug Dis..

[12] Tiihonen K, Ouwehand AC, Rautonen N (2010). Human intestinal microbiota and healthy ageing. Age. Res. Rev..

[13] Park S-Y, Lee J-W, Lim S-D (2014). The probiotic characteristics and GABA production of Lactobacillus plantarum K154 isolated from kimchi. Food Sci. Biotechnol..

[14] Mohanty, D., Saini, M. R. & Mohapatra, S. In vitro study on release of bioactive antimicrobial compounds from dairy products by certain promising probiotic lactobacillus strains. *International Journal of Pharmacy and Pharmaceutical Sciences*, 27–31 (2017).

[15] Bharti V (2015). Bacteriocin: a novel approach for preservation of food. Int. J. Pharm. Pharm. Sci.

[16] Diez-Gutiérrez L, San Vicente L, Barrón LJR, del Carmen Villarán M, Chávarri M (2020). Gamma-aminobutyric acid and probiotics: Multiple health benefits and their future in the global functional food and nutraceuticals market. J. Func. Foods.

[17] Kumaree KK, Akbar A, Anal AK (2015). Bioencapsulation and application of Lactobacillus plantarum isolated from catfish gut as an antimicrobial agent and additive in fish feed pellets. Ann. Microbiol..

[18] Miniello V, Diaferio L, Lassandro C, Verduci E (2017). The importance of being eubiotic. J. Prob. Health.

[19] Claesson MJ (2011). Composition, variability, and temporal stability of the intestinal microbiota of the elderly. Proc. Natl. Acad. Sci..

[20] O’Toole PW, Jeffery IB (2015). Gut microbiota and aging. Science.

[21] Niccoli T, Partridge L (2012). Ageing as a risk factor for disease. Curr. Biol..

[22] López-Otín C, Blasco MA, Partridge L, Serrano M, Kroemer G (2013). The hallmarks of aging. Cell.

[23] Alsegiani AS, Shah ZA (2022). The influence of gut microbiota alteration on age-related neuroinflammation and cognitive decline. Neural Regen. Res..

[24] Bourassa MW, Alim I, Bultman SJ, Ratan RR (2016). Butyrate, neuroepigenetics and the gut microbiome: Can a high fiber diet improve brain health?. Neurosci. Lett..

[25] Facciotti F (2022). Modulation of intestinal immune cell responses by eubiotic or dysbiotic microbiota in inflammatory bowel diseases. PharmaNutrition.

[26] Odamaki T (2016). Age-related changes in gut microbiota composition from newborn to centenarian: a cross-sectional study. BMC Microbiol..

[27] Biagi E (2010). Through ageing, and beyond: gut microbiota and inflammatory status in seniors and centenarians. PloS one.

[28] Naomi R (2021). Probiotics for Alzheimer’s disease: a systematic review. Nutrients.

[29] Cattaneo A (2017). Association of brain amyloidosis with pro-inflammatory gut bacterial taxa and peripheral inflammation markers in cognitively impaired elderly. Neurobiol. Aging.

[30] Vogt NM (2017). Gut microbiome alterations in Alzheimer’s disease. Sci. Rep..

[31] Bonfili L (2017). Microbiota modulation counteracts Alzheimer’s disease progression influencing neuronal proteolysis and gut hormones plasma levels. Sci. Rep..

[32] Kobayashi Y (2017). Therapeutic potential of Bifidobacterium breve strain A1 for preventing cognitive impairment in Alzheimer’s disease. Sci. Rep..

[33] Jones KT, Ashrafi K (2009). Caenorhabditis elegans as an emerging model for studying the basic biology of obesity. Dis. Mode. Mech..

[34] Sobkowiak, R., Kaczmarek, P., Kowalski, M., Kabaciński, R. & Lesicki, A. Caenorhabditis elegans as an emerging model for studying the basic biology of anorectic effects of nicotine. *bioRxiv*, 099952 (2017).

[35] Schulenburg H, Léopold Kurz C, Ewbank JJ (2004). Evolution of the innate immune system: the worm perspective. Immunol. Rev..

[36] Montalvo-Katz S, Huang H, Appel MD, Berg M, Shapira M (2013). Association with soil bacteria enhances p38-dependent infection resistance in Caenorhabditis elegans. Infect. Immun..

[37] Prasanth, M. I., Brimson, J. M., Chuchawankul, S., Sukprasansap, M. & Tencomnao, T. Antiaging, stress resistance, and neuroprotective efficacies of Cleistocalyx nervosum var. paniala fruit extracts using Caenorhabditis elegans model. *Oxidat. Med. Cell. Longev.* **2019** (2019). 10.1155/2019/7024785 PMC6906846 31871554

[38] Brimson, J. M. *et al.* Bacopa monnieri protects neuronal cell line and Caenorhabditis elegans models of Alzheimer’s disease through sigma-1 receptor antagonist sensitive and antioxidant pathways. *Nutr. Healthy Aging*, 1–24.

[39] Chakravarty, B. The evolving role of the Caenorhabditis elegans model as a tool to advance studies in nutrition and health. *Nutr. Res.* (2022). 10.1016/j.nutres.2022.05.006 36126529

[40] Mahesh R, Ilangovan P, Nongbri D, Suchiang K (2021). Probiotics interactions and the modulation of major signalling pathways in host model organism caenorhabditis elegans. Indian J. Microbiol..

[41] Wang S (2020). Lipoteichoic acid from the cell wall of a heat killed Lactobacillus paracasei D3–5 ameliorates aging-related leaky gut, inflammation and improves physical and cognitive functions: from C. elegans to mice. Geroscience.

[42] de Barros PP (2018). Lactobacillus paracasei 28.4 reduces in vitro hyphae formation of Candida albicans and prevents the filamentation in an experimental model of Caenorhabditis elegans. Microb. Pathogen..

[43] Grompone G (2012). Anti-inflammatory Lactobacillus rhamnosus CNCM I-3690 strain protects against oxidative stress and increases lifespan in Caenorhabditis elegans. PloS one.

[44] Kumar A (2022). A potential probiotic Lactobacillus plantarum JBC5 improves longevity and healthy aging by modulating antioxidative, innate immunity and serotonin-signaling pathways in Caenorhabditis elegans. Antioxidants.

[45] Chaiyasut C (2021). Effect of Lactobacillus paracasei HII01 supplementation on total cholesterol, and on the parameters of lipid and carbohydrate metabolism, oxidative stress, inflammation and digestion in Thai hypercholesterolemic subjects. Appl. Sci..

[46] Lalitsuradej E (2022). The effects of synbiotics administration on stress-related parameters in thai subjects—a preliminary study. Foods.

[47] Perelman A (2012). JC-1: alternative excitation wavelengths facilitate mitochondrial membrane potential cytometry. Cell Death Dis..

[48] Claesson MJ (2012). Gut microbiota composition correlates with diet and health in the elderly. Nature.

[49] Williams NT (2010). Probiotics. Am. J. Health-Syst. Pharm..

[50] Cryan, J. F. *et al.* The microbiota-gut-brain axis. *Physiol. Rev.* (2019). 10.1152/physrev.00018.2018 31460832

[51] Ikeda T, Yasui C, Hoshino K, Arikawa K, Nishikawa Y (2007). Influence of lactic acid bacteria on longevity of Caenorhabditis elegans and host defense against Salmonella enterica serovar enteritidis. Appl. Environ. Microbiol..

[52] Kwon G, Lee J, Lim Y-H (2016). Dairy Propionibacterium extends the mean lifespan of Caenorhabditis elegans via activation of the innate immune system. Sci. Rep..

[53] Chen Y (2020). Transplant of microbiota from long-living people to mice reduces aging-related indices and transfers beneficial bacteria. Aging (Albany NY).

[54] Kim S, Jazwinski SM (2018). The gut microbiota and healthy aging: a mini-review. Gerontology.

[55] Kim Y, Mylonakis E (2012). Caenorhabditis elegans immune conditioning with the probiotic bacterium Lactobacillus acidophilus strain NCFM enhances gram-positive immune responses. Infect. Immun..

[56] Libina N, Berman JR, Kenyon C (2003). Tissue-specific activities of C. elegans DAF-16 in the regulation of lifespan. Cell.

[57] Oh SW (2005). JNK regulates lifespan in Caenorhabditis elegans by modulating nuclear translocation of forkhead transcription factor/DAF-16. Proc. Natl. Acad. Sci..

[58] Kumar, S., Praneet, N. S. & Suchiang, K. Lactobacillus brevis MTCC 1750 enhances oxidative stress resistance and lifespan extension with improved physiological and functional capacity in Caenorhabditis elegans via the DAF-16 pathway. *Free Rad. Res.* 1–41 (2022). 10.1080/10715762.2022.2155518 36480684

[59] Bratic A, Larsson N-G (2013). The role of mitochondria in aging. J. Clin. Invest..

[60] Xiao Y (2021). Human gut-derived B. longum subsp. longum strains protect against aging in ad-galactose-induced aging mouse model. Microbiome.

[61] Moreno-Arriola, E. *et al.* Caenorhabditis elegans: A useful model for studying metabolic disorders in which oxidative stress is a contributing factor. *Oxidative medicine and cellular longevity* **2014** (2014). 10.1155/2014/705253 PMC4052186 24955209

[62] Lemire BD, Behrendt M, DeCorby A, Gášková DC (2009). elegans longevity pathways converge to decrease mitochondrial membrane potential. Mech. Age. Dev..

[63] Baumeister R, Schaffitzel E, Hertweck M (2006). Endocrine signaling in Caenorhabditis elegans controls stress response and longevity. J. Endocrinol..

[64] Torgovnick A, Schiavi A, Maglioni S, Ventura N (2013). Healthy aging: what can we learn from Caenorhabditis elegans? Gesundes Altern: Was können wir von Caenorhabditis elegans lernen?. Zeitschrift für Gerontologie und Geriatrie.

[65] Kapahi P, Kaeberlein M, Hansen M (2017). Dietary restriction and lifespan: Lessons from invertebrate models. Age. Res. Rev..

[66] Lapierre LR, Hansen M (2012). Lessons from C. elegans: signaling pathways for longevity. Trends Endocrinol. Metabol..

[67] Roselli M (2019). Caenorhabditis elegans and probiotics interactions from a prolongevity perspective. Int. J. Mol. Sci..

[68] Garigan D (2002). Genetic analysis of tissue aging in Caenorhabditis elegans: a role for heat-shock factor and bacterial proliferation. Genetics.

[69] Papp D, Csermely P, Sőti C (2012). A role for SKN-1/Nrf in pathogen resistance and immunosenescence in Caenorhabditis elegans. PLoS Pathogens.

[70] Kayser E-B, Sedensky MM, Morgan PG, Hoppel CL (2004). Mitochondrial oxidative phosphorylation is defective in the long-lived mutant clk-1. J. Biol. Chem..

[71] Du F (2019). Ingredients in Zijuan Pu’er tea extract alleviate β-amyloid peptide toxicity in a Caenorhabditis elegans model of Alzheimer’s disease likely through DAF-16. Molecules.

[72] Brenner S (1974). The genetics of Caenorhabditis elegans. Genetics.

[73] Zhou M (2018). Cell signaling of Caenorhabditis elegans in response to enterotoxigenic Escherichia coli infection and Lactobacillus zeae protection. Front. Immunol..

[74] Chelliah R (2018). In vitro and in vivo defensive effect of probiotic LAB against Pseudomonas aeruginosa using Caenorhabditis elegans model. Virulence.

[75] Nakagawa H (2016). Effects and mechanisms of prolongevity induced by Lactobacillus gasseri SBT2055 in Caenorhabditis elegans. Aging cell.

[76] Livak KJ, Schmittgen TD (2001). Analysis of relative gene expression data using real-time quantitative PCR and the 2− ΔΔCT method. Methods.

