# Supplementary material for: Lactobacillus paracasei HII01 enhances lifespan and promotes neuroprotection in Caenorhabditis elegans
Source: Sci Rep. 2023 Oct 4;13:16707. doi: 10.1038/s41598-023-43846-9 (PMC10550917; doi:10.1038/s41598-023-43846-9)
Supplement: Supplementary file 1 — Supplementary Information. [file 41598_2023_43846_MOESM1_ESM.docx]

**Supplementary Material**

***Lactobacillus paracasei* HII01 enhances lifespan and promotes neuroprotection in *Caenorhabditis elegans***

**(a)**


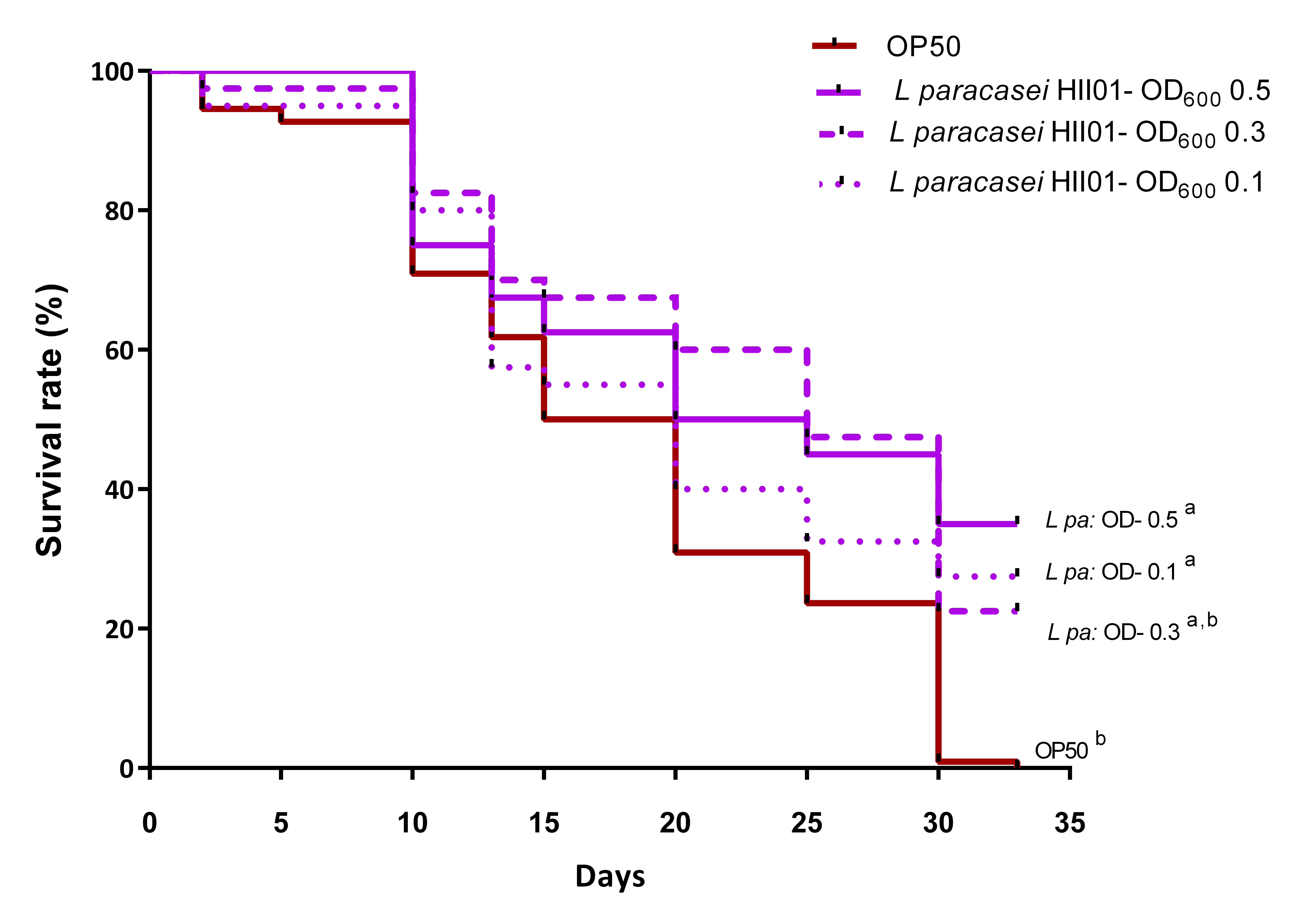


**(b)**

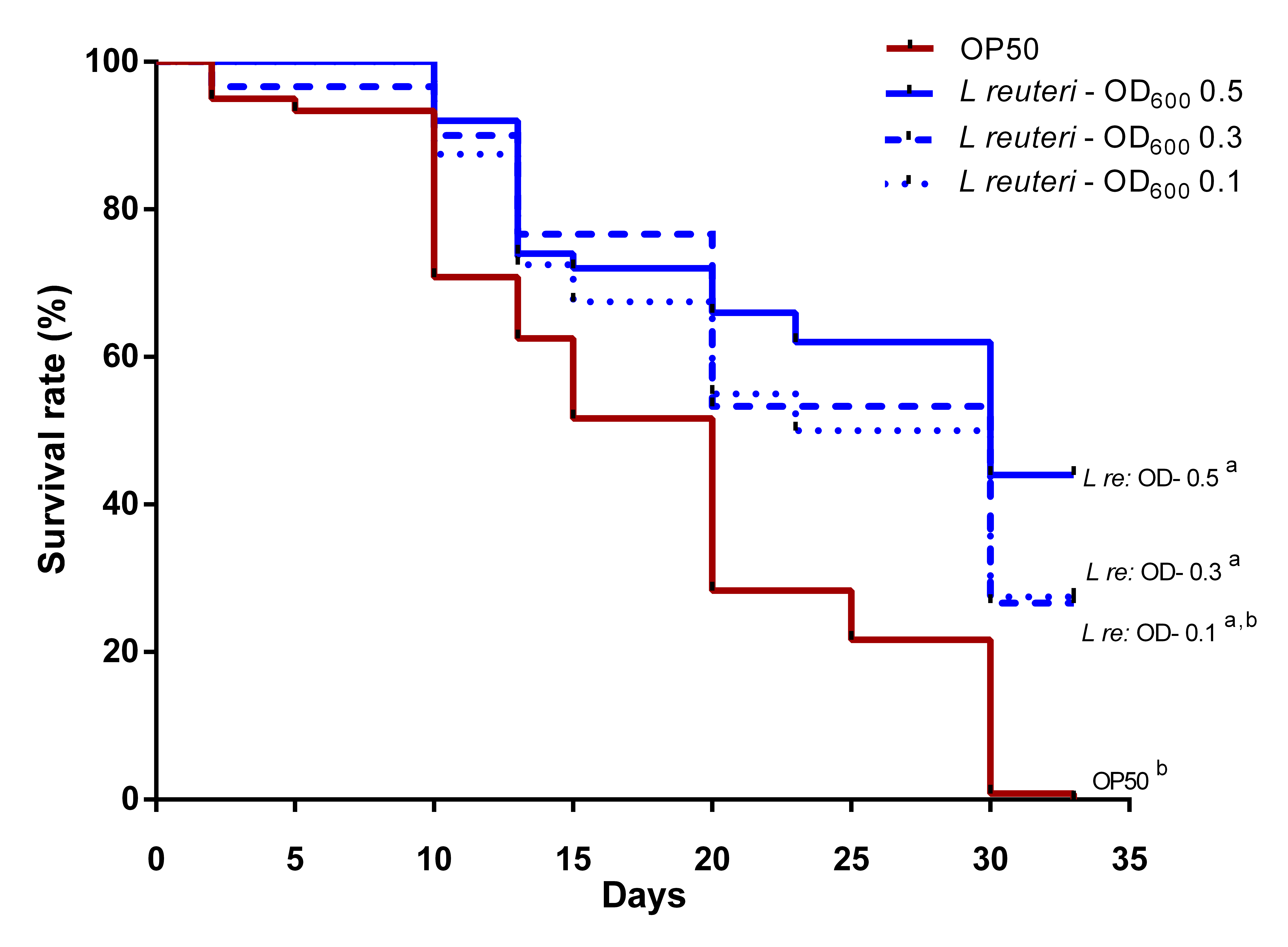


**(c)**

**(d)**


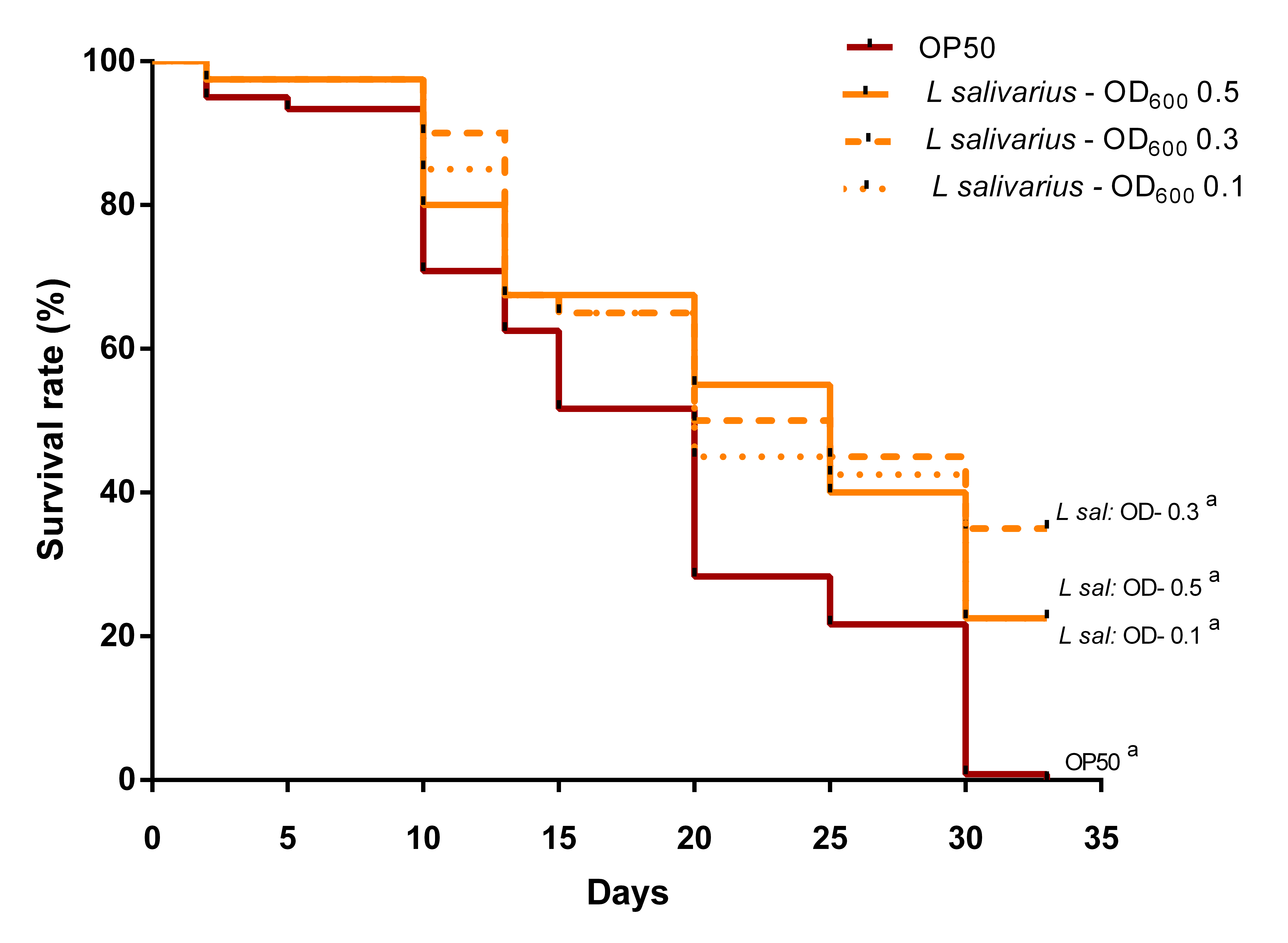


**Supplementary Figure S1.** Kaplan-Meier survival curve of wild type *C. elegans* fed on four different LAB strains separately or *E. coli* OP50. Three different doses of all LAB strains were diluted based on optical density (OD_600_); doses tested for the study were OD_600_ – 0.5. 0.3 and 0.1. **(a)** *L. paracasei* HII01, **(b)** *L. rhamnosus*, **(c)** *L reuteri*, **(d)** *L salivarius.* All the groups showing different letters were significantly different (*p* < 0.05) in their survival curves.

**Supplementary Figure S2.** Bacterial colonization ability of four different LAB strains in *C. elegans*. Investigating the colonization ability of our four LAB strains (OD_600_ 0.5) in the intestine of the nematodes. Bar graph showing one-way ANOVA for the CFU/ml (mean ± SEM). All the groups showing different letters in their superscript were significantly different (*p* < 0.01).

**Supplementary Table S1.** Primer sequences of the genes used in the qPCR analysis.

| Gene name | Forward primer (5’ 🡪 3’) | Reverse Primer (5’ 🡪 3’) |
| --- | --- | --- |
| *act-2* | ATCGTCCTCGACTCTGGAGATG | TCACGTCCAGCCAAGTCAAG |
| *age-1* | AGTGGATTCGGAAACAATGC | GGAATCGATCGACACTTTCA |
| *clk-1* | GCAATAGCTCCCTTGCATCC | AGCACATACTGCTGCTTCTC |
| *daf-2* | TCGAGCTCTTCCTACGGTGT | CATCTTGTCCACCACGTGTC |
| *daf-16* | TTTCCGTCCCCGAACTCAA | ATTCGCCAACCCATGATGG |
| *hsf-1* | GCGGCTCCGTATAAGAATGCGACTAGGC | TTAAACCAAATTAGGATCCGATGGACTTGGAGTAC |
| *nsy-1* | AGTTGTAGACACGCCGGTTA | TGCACTGTTCAGGCTTTCAC |
| *pmk-1* | CCGACTCCACGAGAAGGATA | AGCGAGTACATTCAGCAGCA |
| *sir-2.1* | CGGGGAAGTGCAAGAAATAA | GAGTGGCACCATCATCAAGA |
| *skn-1* | ATCCATTCGGTAGAGGACCA | GGCGCTACTGTCGATTTCTC |
| *utx-1* | GCAGAACACCAGCTCATCAG | ATCAACGCCATTCTTCTCGC |

**Supplementary Table S2.** *C. elegans* genes involved in the aging process and their mammalian orthologs.

| *C. elegans*  gene name | Description/ mammalian ortholog gene name |
| --- | --- |
| *daf-2* | Insulin/IGF-I receptor |
| *age-1* | Phosphatidylinositol-3-kinase (PI3K) p110 catalytic subunit |
| *daf-16* | Forkhead transcription factor (FOXO3A) |
| *clk-1* | CLK1/COQ7, biosynthesis of ubiquinone |
| *skn-1* | bZIP transcription factor NRF1 |
| *utx-1* | UTX/KDM6A |
| *pmk-1* | p38 mitogen-activated protein kinase (MAPK) |
| *nsy-1* | MAPK kinase kinase (MAPKKK) |
| *hsf-1* | Heat shock transcription factor 2 |
| *sir-2.1* | NADC-dependent histone deacetylase |
| *act-2* | Actin |
